# Monitoring and Evaluating the Quality Consistency of Compound Bismuth Aluminate Tablets by a Simple Quantified Ratio Fingerprint Method Combined with Simultaneous Determination of Five Compounds and Correlated with Antioxidant Activities

**DOI:** 10.1371/journal.pone.0118223

**Published:** 2015-03-20

**Authors:** Yingchun Liu, Zhongbo Liu, Guoxiang Sun, Yan Wang, Junhong Ling, Jiayue Gao, Jiahao Huang

**Affiliations:** 1 Key Laboratory of Structure-Based Drug Design and Discovery, Ministry of Education, School of Pharmaceutical Engineering, Shenyang Pharmaceutical University, Shenyang, China; 2 Pharmaceutical Informatics Laboratory, School of Pharmacy, Shenyang Pharmaceutical University, Shenyang, China; Aligarh Muslim University, INDIA

## Abstract

A combination method of multi-wavelength fingerprinting and multi-component quantification by high performance liquid chromatography (HPLC) coupled with diode array detector (DAD) was developed and validated to monitor and evaluate the quality consistency of herbal medicines (HM) in the classical preparation Compound Bismuth Aluminate tablets (CBAT). The validation results demonstrated that our method met the requirements of fingerprint analysis and quantification analysis with suitable linearity, precision, accuracy, limits of detection (LOD) and limits of quantification (LOQ). In the fingerprint assessments, rather than using conventional qualitative “Similarity” as a criterion, the simple quantified ratio fingerprint method (SQRFM) was recommended, which has an important quantified fingerprint advantage over the “Similarity” approach. SQRFM qualitatively and quantitatively offers the scientific criteria for traditional Chinese medicines (TCM)/HM quality pyramid and warning gate in terms of three parameters. In order to combine the comprehensive characterization of multi-wavelength fingerprints, an integrated fingerprint assessment strategy based on information entropy was set up involving a super-information characteristic digitized parameter of fingerprints, which reveals the total entropy value and absolute information amount about the fingerprints and, thus, offers an excellent method for fingerprint integration. The correlation results between quantified fingerprints and quantitative determination of 5 marker compounds, including glycyrrhizic acid (GLY), liquiritin (LQ), isoliquiritigenin (ILG), isoliquiritin (ILQ) and isoliquiritin apioside (ILA), indicated that multi-component quantification could be replaced by quantified fingerprints. The Fenton reaction was employed to determine the antioxidant activities of CBAT samples *in vitro*, and they were correlated with HPLC fingerprint components using the partial least squares regression (PLSR) method. In summary, the method of multi-wavelength fingerprints combined with antioxidant activities has been proved to be a feasible and scientific procedure for monitoring and evaluating the quality consistency of CBAT.

## Introduction

According to statistics, approximately 10% of the world population suffers from gastric ulcers [1], which are caused or aggravated by etiological factors including infection of *Helicobacter pylori*, stress, frequent use of nonsteroidal anti-inflammatory drugs, smoking and alcohol consumption [2]. CBAT, a popular medicine used around the world, has been used for over 20 years in the treatment of such ulcers. CBAT includes not only three synthetic drugs, bismuth aluminate, heavy magnesium carbonate and sodium hydrogen carbonate, but also three medicinal herbs, Extract Licorice, Cortex Frangulae and Fructus Foeniculi (300, 25 and 10 mg in one tablet, respectively) [3]. Licorice, as a principal herb in CBAT, mainly contains triterpene saponins and flavonoids, which have been suggested to be main bioactive constituents [4,5]. Such major bioactive components as GLY, LQ, ILQ, ILG, ILA, etc. were often selected as marker compounds for the purpose of Licorice quality control [6–8].

Due to the mystery of chemical composition of TCM/HM that varies depending on a wide range of factors, such as the botanical species, the anatomical part of the plant used, the amount of sun the plant has been exposed to, the type of ground in which it is grown, harvesting time, cultivating region, and storage conditions [9], it is very difficult to maintain the quality consistency of TCM/HM at both manufacturer and batch level. In addition, TCM/HM are composed of hundreds of chemically different constituents, and multiple compounds often work synergistically in delivering therapeutic effects [10]. Consequently, a comprehensive and effective quality control method capable of guaranteeing their safety and efficacy in clinical applications is absolutely imperative.

Now CBAT has been documented in the Chinese Pharmacopoeia (2010) and a quantitative standard with respect to its HM only involves GLY. Other reports concerning CBAT have mainly focused on analytical methods for bismuth [11–15] and have been confined to quantification of a single bioactive component [16,17]. The fingerprinting technique with an overall view of a complicated system has been proved to be of value in determining the identity, authenticity and batch-to-batch consistency of TCM/HM. Hence, such analytical profiles have been accepted and adopted as a strategy for TCM/HM quality assessment by the Food and Drug Administration (FDA) of the USA [18], European Medicines Agency (EMA) [19] and the State Food and Drug Administration (SFDA) of China [20,21]. Fingerprinting can be carried out using both chromatographic and spectroscopic techniques [22–27], and the chromatographic approach, especially HPLC, is regarded as one of the most potential and reliable means for TCM/HM quality control.

**Fig 1:**
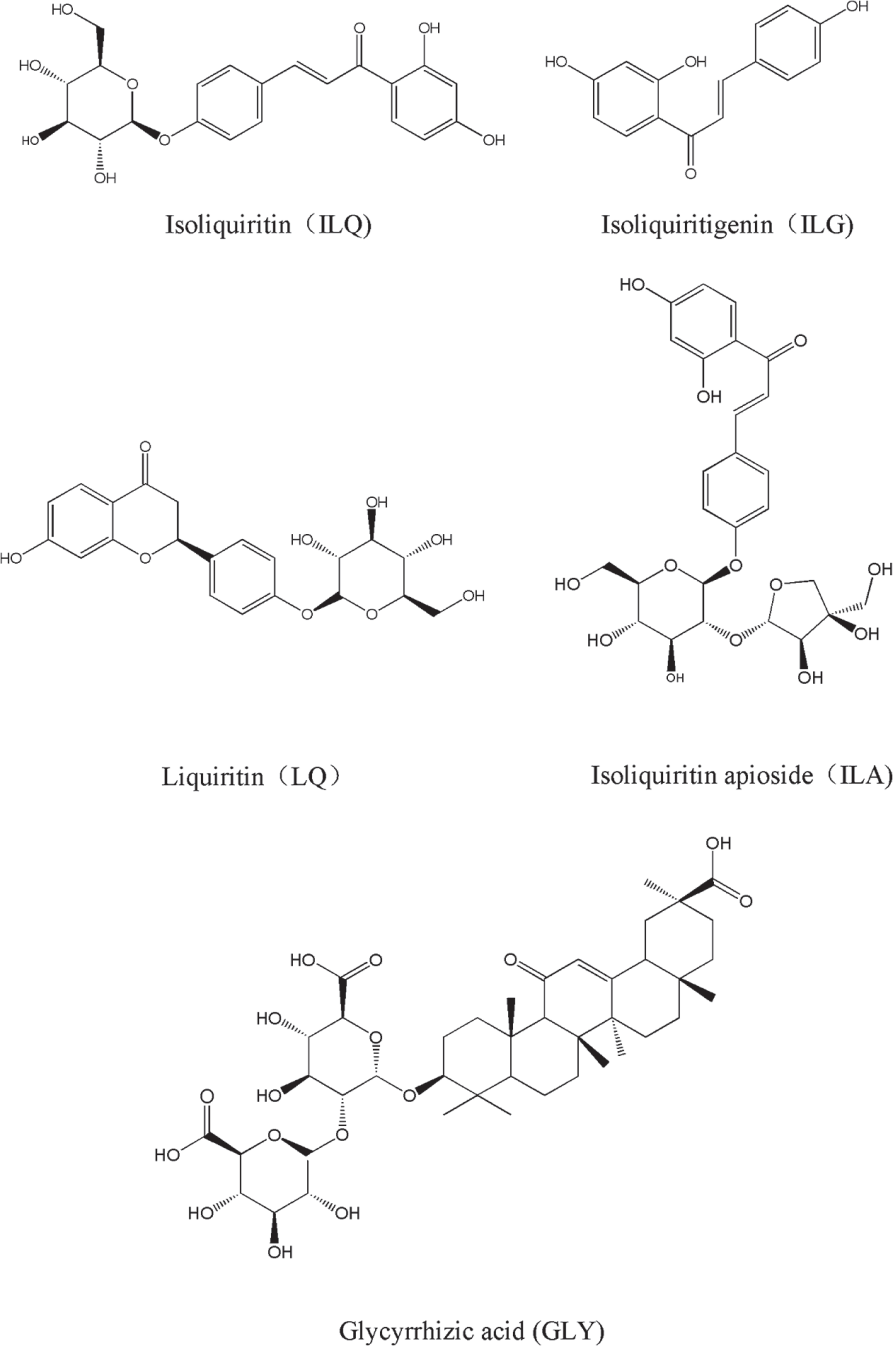
The molecular structures of the 5 marker compounds.

In the present study, we strategically established multi-wavelength chromatographic fingerprints and simultaneously determined 5 marker compounds to assess the HM quality in CBAT by HPLC-DAD, and the molecular structures of the quantification involved are shown in Fig. 1. In the fingerprint assessments, SQRFM, which has an outstanding quantified fingerprint advantage and exhibits a clear TCM/HM quality pyramid and warning gate using the combination of $S_{\text{F}}^{\prime }$, *M*_F_ and *α*, was employed to assess the HM quality grades from a both qualitative and quantitative perspective. In order to combine the comprehensive characterization of multi-wavelength ratio fingerprint profiles, an integrated fingerprint assessment method based on information entropy was set up. Because CBAT contains many components with antioxidant activities, correlation analysis between chromatographic fingerprint components and antioxidant activities of CBAT samples were performed and we obtained a robust calibration model by PLSR, which provided important medicinal efficacy information *in vitro* for CBAT quality control. It has been demonstrated that the present study offered an efficient way to monitor and evaluate the quality consistency of CBAT.

## Theory of SQRFM

**Fig 2:**
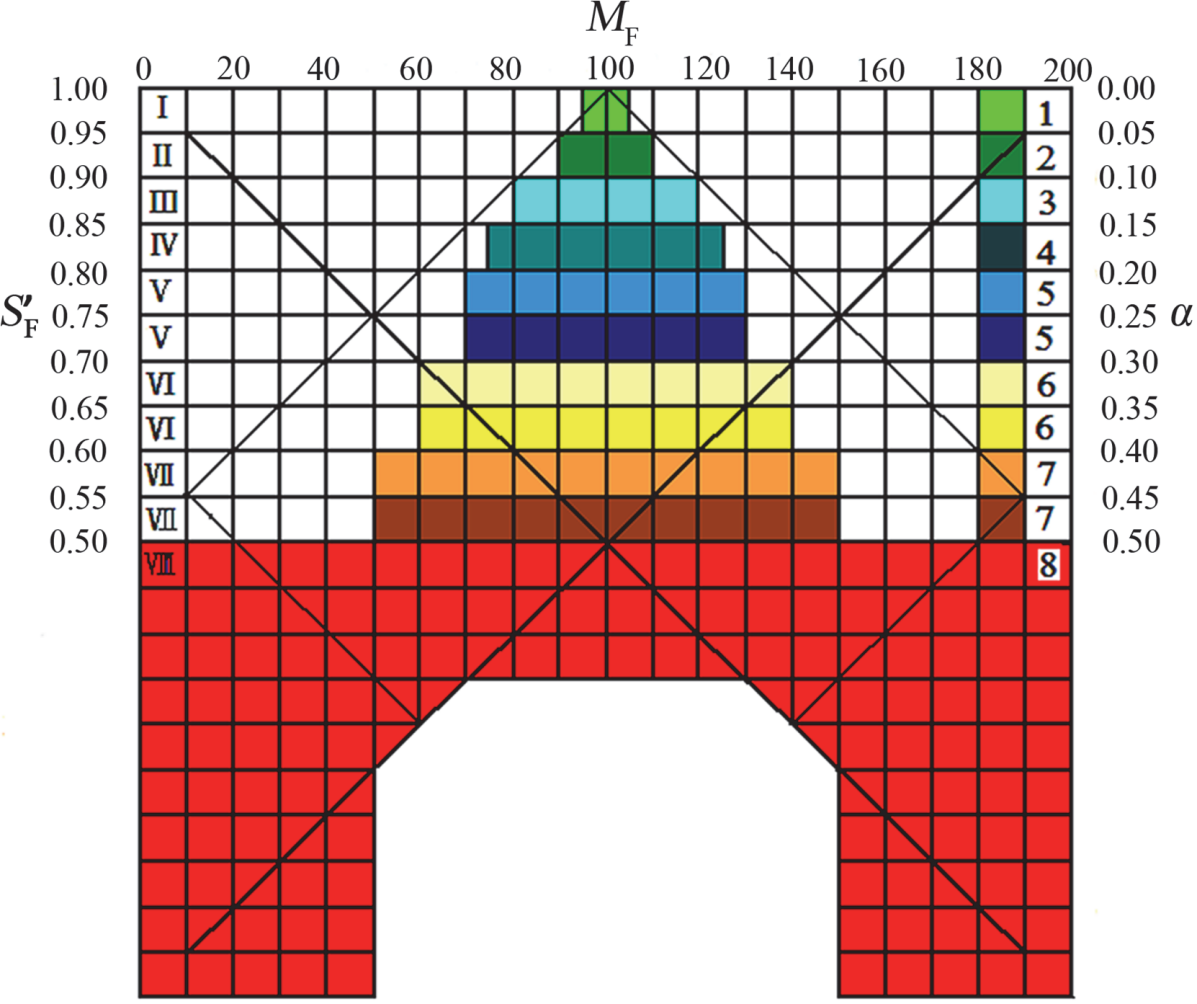
The quality pyramid and warning gate for TCM/HM based on SQRFM. $S_{\text{F}}^{\prime }$: the qualitative ratio similarity; *M*_F_: the corrected quantified ratio similarity; *α*: the coefficient of variation.

In the sample fingerprint vector (SFPV) $\vec{x}=(x_{1},x_{2},\ldots ,x_{n})$ and the reference fingerprint vector (RFPV) $\vec{y}=(y_{1},y_{2},\ldots ,y_{n})$, *x*_*i*_ and *y*_*i*_ represent the peak area of the *i*th constituent in the sample and reference fingerprints, respectively. The ‘Similarity’, *S*_F_, between SFPV and RFPV can be expressed by Eq (1), as recommended by SFDA [28]. Moreover, the ratio similarity, $S_{\text{F}}^{\prime }$, in the ratio fingerprint profile as defined in Eq (2) can effectively cancel the effect of larger peaks masking smaller peaks [29]. A ratio fingerprint profile can be obtained by plotting *x*_*i*_/*y*_*i*_ vs. the retention time, meanwhile, SFPV and RFPV are converted into $\vec{X_{\text{r}}}=(x_{1}/y_{1},x_{2}/y_{2},\cdots ,x_{n}/y_{n})$ and $\vec{Y_{\text{r}}}=(1,1,\cdots ,1)$, respectively. $S_{\text{F}}^{\prime }$ not only clearly demonstrates the resemblance in the number and distribution of fingerprint peaks between SFPV and RFPV, but also takes into consideration the major and minor peaks in equal weights. The mean total content (*M*) of all the components in the ratio fingerprint profile can be calculated by Eq (3) and its corrected model, which can eliminate the error from cross-compensation among *x*_*i*_/*y*_*i*_ values, is shown in Eq (4), where *M*_F_ represents the corrected quantified ratio similarity. It should be noted that *M*_F_ is also corrected for sample weight using a mass coefficient (*f*_*i*_), which is defined as the ratio of the apparent weight of RFP (*m*_R_) and the weight of the *i*th sample (*m*_*i*_). The coefficient of variation (*α*) is defined in Eq (5) to reflect the dissimilarity between them. The approach to assess TCM/HM quality using the combination of $S_{\text{F}}^{\prime }$, *M*_F_ and *α* in ratio fingerprint profiles is called SQRFM, by which TCM/HM are divided into 8 grades (Table 1). In the evaluation system, the TCM/HM quality pyramid and warning gate are clearly presented in Fig. 2. At the top of the pyramid is grade 1 which is the highest quality, so the proper criteria falling into grade 1–5 are certainly considered as qualified. The whole red region is termed the quality warning gate which reveals the characteristics of adulterants with $S_{\text{F}}^{\prime }$ < 0.5, *α* > 0.50 and any *M*_F_.

$$S_{\text{F}}=\frac{\sum_{i=1}^{n}x_{i}y_{i}}{\sqrt{\sum_{i=1}^{n}x_{i}^{2}}\sqrt{\sum_{i=1}^{n}y_{i}^{2}}}$$ (1)

$${S^{\prime }}_{\text{F}}^{}=\frac{\sum_{i=1}^{n}\frac{x_{i}}{y_{i}}}{\sqrt{n\sum_{i=1}^{n}{\left(\frac{x_{i}}{y_{i}}\right)}^{2}}}$$ (2)

$$S_{m}=\frac{1}{2}\times \left(S_{F}+S_{F}^{`}\right)=\frac{1}{2}(\frac{\sum_{i=1}^{n}x_{i}y_{i}}{\sqrt{\sum_{i=1}^{n}{xi}^{2}\sum_{i=1}^{n}{yi}^{2}}}+\frac{\sum_{i=1}^{n}\frac{x_{i}}{y_{i}}}{\sqrt{n\sum_{i=1}^{n}{\left(\frac{x_{i}}{y_{i}}\right)}^{2}}})M=\frac{1}{n}\sum_{i=1}^{n}\frac{x_{i}}{y_{i}}\times 100\%$$ (3)

$$M_{\text{F}}=MS_{\text{F}}^{\prime }=\frac{1}{n}\sum_{i=1}^{n}\frac{x_{i}}{y_{i}}\cdot S_{\text{F}}^{\prime }\cdot f_{i}\times 100\%$$ (4)

$$\alpha =\left|1-S_{\text{F}}^{\prime }\right|$$ (5)

**Table 1:** The TCM/HM quality grades classified by SQRFM.

Parameter	1	2	3	4	5	6	7	8
$S_{\text{F}}^{\prime }$≥	0.95	0.9	0.85	0.8	0.7	0.6	0.5	*$S_{\text{F}}^{\prime }<0.5$*
*M*_F_% **ε**	95–105	90–110	80–120	75–125	70–130	60–140	50–150	0–∞
***α* ≤**	0.05	0.1	0.15	0.2	0.3	0.4	0.5	*α* > 0.50
**Quality grade**	1	2	3	4	5	6	7	8

## Materials and Methods

### Reagents and Chemicals

Standards, including ILQ, ILG, LQ, ILA and glycyrrhizic acid ammonium salt (GHIA), were supplied by Winherb Science and Technology Inc. (Shanghai, China). Acetonitrile, methanol and glacial acetic acid (HPLC grade), as well as ferrous sulfate heptahydrate (FeSO_4_·7H_2_O), 30% hydrogen peroxide (H_2_O_2_) and crystal violet (analytical grade) were all purchased from Yuwang Industry Co., Ltd. (Shandong, China). Deionized water was used for all the experiments. Commercial products of CBAT were manufactured by Tonglian Pharmaceutical Co., Ltd. (Shenyang, China) (The Batch No. of 27 drugs numbered S1–S27 were 110905, 1201063, 120203, 120801, 1201054, 1204003, 1202033, 1201061, 1201062, 1201056, 1201053, 1201052, 1202044, 1203094, 1201057, 1201063, 120503, 120531, 120306, 120526, 120515, 120706, 110102, 111126, 120327, 120709 and 1201060, respectively.).

### Instruments

Chromatographic analysis was carried out on an Agilent 1100 HPLC series (Agilent Technologies, USA) equipped with a G1315B UV–vis DAD, a G1311A low pressure quaternary pump, a G1379A on-line degasser and a G1313A auto-sampler. System control and data analysis were carried out using an Agilent ChemStation workstation (Agilent, USA). Two columns, a Century SIL C_18_ BDS (250 × 4.6 mm; 5.0 μm) (Century, China) and an Agilent poroshell 120SB C_18_ (150 × 4.6 mm; 2.7 μm) (Agilent, USA), were used to optimize the separation conditions. The instrument for antioxidant activity assay was a 752–UV spectrophotometer (Gaomi Caihong, China).

### Sample and Standard Solution Pretreatment

The tested samples were ground into powder with a mortar. Each powder sample (1.2 g) was accurately weighed and extracted three times with 25 ml of methanol for half an hour by refluxing. The combined extracts were concentrated in a rotary evaporator under vacuum, and then diluted to the mark with methanol in a 25 ml volumetric flask. The solutions were kept at 4˚C.

Standard stock solutions of the 5 standards, ILQ, ILG, LQ, ILA and GHIA, were accurately weighed and then dissolved in methanol. A mixed standard solution was prepared by mixing the 5 individual standard stock solutions, and then six concentration levels of mixed standard solutions for the calibration curves were obtained by diluting the above mixed standard solution with methanol. These standard solutions were kept at 4˚C.

All the sample and standard solutions were filtered using a 0.22 μm nylon membrane prior to HPLC analysis.

All the sample fingerprint profiles were analyzed and evaluated by independently developed software ‘Digitized Evaluation System for Super-Information Characteristics of TCM/HM Chromatographic Fingerprints 4.0’ (Software certificate No. 0407573, China). In addition, SPSS 19.0, MATLAB 2009a, ORIGIN 8.5 and SIMCA 13.0 were also used for data processing and drawing.

### Chromatographic Condition

The Century SIL C_18_ BDS column (250 × 4.6 mm; 5 μm) was used for chromatographic system. The mobile phase was composed of water-glacial acetic acid (A; 100:1, v/v) and acetonitrile-glacial acetic acid (B; 100:1, v/v). The linear gradient program was set as follows: 5–8% B over 0–8 min; 8–25% B over 8–25 min; 25–30% B over 25–45 min; 30–50% B over 45–65 min; 50–70% B over 65–85 min; 70–77% B over 85–90 min. The effluent from the column was detected by a DAD and the detection wavelengths were set at 250, 276, 330, 360 and 375 nm. The flow rate, column temperature and loading volume were kept at 0.5 ml/min, 35°C and 5 μl, respectively.

### Determination of Antioxidant Activity

$${\text{H}}_{\text{2}}{\text{O}}_{\text{2}}+{\text{ Fe}}^{\text{2}+}\to {\text{Fe}}^{\text{3}+}+{\text{ OH}}^{-}+\text{OH}\cdot$$ (6)

We used the Fenton reaction [30] to determine the antioxidant activity of CBAT. Ferrous irons react with H_2_O_2_ in an acidic medium to generate hydroxyl radicals (OH•) as shown in Eq (6); they possess outstanding oxidant ability and induce an advanced oxidation reaction, causing fading of the crystal violet. After adding the antioxidant to eliminate OH•, the degree of crystal violet fading was reduced. The scavenging ratio of OH• was indirectly deduced from the absorbance values of crystal violet.

The blank sample solution was prepared by adding successively 6.0 ml of 1.0 mmol/L FeSO_4_·7H_2_O solution, 1.0 ml of 0.4 mmol/L crystal violet solution, and 0.09 ml of potassium hydrogen phthalate buffer solution (pH = 4.0) to a 25 ml volumetric flask and diluting to the mark with water, and then the absorbance (*A*_0_) of the blank sample solution was measured at 588 nm. The reaction solution was prepared by adding 1.1 ml of 1% H_2_O_2_ solution to the blank sample solution, then the absorbance (*A*_b_) was measured.

The negative control samples were prepared by adding different volumes of methanol (0.10, 0.20, 0.25, 0.50 and 0.75 ml) to the blank sample solution, and the absorbance values (*A*_B_) were measured. Finally the positive control samples were prepared by replacing methanol with corresponding volumes of sample solution as described in the section of ‘Sample and Standard Solution Pretreatment’. These assay solutions were shaken and stored at 4˚C for 30 min, and then the absorbance values (*A*_S_) were measured. The scavenging ratio (SR) of OH• was calculated using Eq (7):

$$\text{SR(}\%\text{)}=\frac{A_{\text{S}}\text{-}A_{\text{B}}}{A_{\text{0}}\text{-}A_{\text{b}}}\times 100\%$$ (7)

where *A*_S_ and *A*_B_ are the absorbance values of the positive control and negative control samples, respectively, *A*_0_ and *A*_b_ are the absorbance values of the blank sample solution without H_2_O_2_ and with H_2_O_2_, respectively. After the added sample volumes were converted into their corresponding concentrations in positive control samples, calibration curves were obtained by plotting SR values vs. sample concentrations, and the sample effective concentration that scavenged 50% of OH• (EC_50_) could be calculated by interpolation.

## Results and Discussion

### Optimization of the Extraction and Chromatography Conditions

In order to obtain as much chemical information as possible and obtain as efficient a chromatographic separation as possible within a short analysis time, the extraction solvents, extraction times, analytical wavelengths, column types and mobile phases for CBAT samples were investigated. The index of the fingerprint information amount (*I*) [31] was applied as an objective function to optimize the extraction and chromatography conditions. The index *I* reflects the total signal strength, the signal homogenization and the information amount in a chromatogram, so the experimental conditions can be assessed by the *I* values of chromatograms and the higher the *I* value the better the condition.

**Fig 3:**
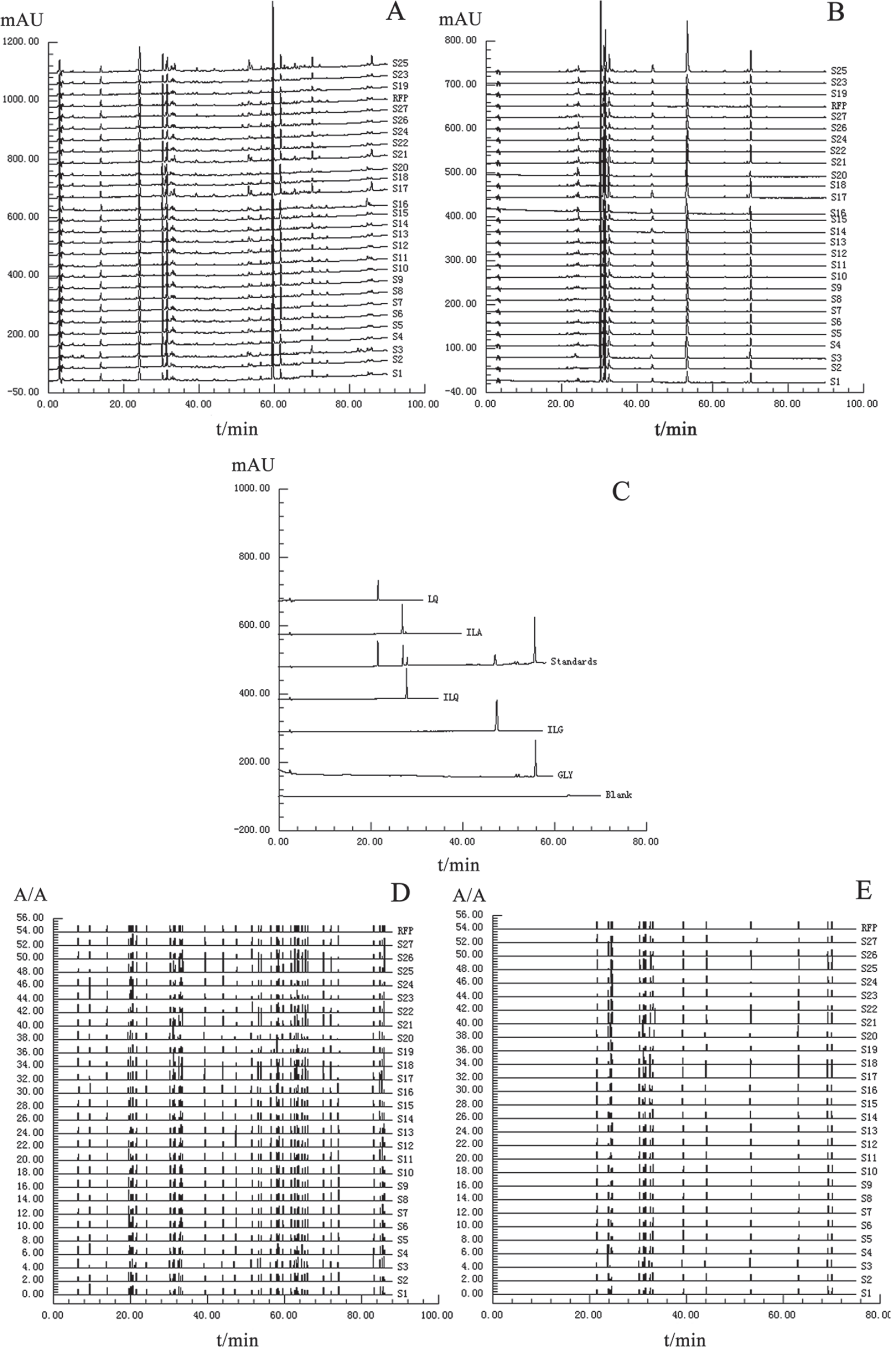
Typical chromatograms and ratio chromatograms of 27 batches of CBAT samples at 5 wavelengths, as well as the chromatograms of marker compound standards. The chromatograms: (A) 250 nm (B) 360 nm (C) standards, the ratio chromatograms: (D) 250 nm (E) 360 nm.

From S1 Fig., it was found that the *I* values of the methanol, 30 min, 250 nm, column 1 and mobile phases 2 were relatively higher than the other conditions, and thus a set of optimized experimental parameters was established as follows: methanol and 30 min were elected as the extraction solvent and extraction time, respectively, and the procedure ‘Sample and Standard Solution Pretreatment’ was adopted; 250 nm, column 1, i.e. the Century SIL C_18_ BDS column (250 × 4.6 mm; 5 μm) as well as mobile phases 2, i.e. water-glacial acetic acid (A; 100:1, v/v) and acetonitrile-glacial acetic acid (B; 100:1, v/v) were used for chromatographic analysis. Because the maximum UV absorbance values of the marker compounds were observed at 250, 276, 330, 360 and 375 nm using a DAD detector, the above 5 wavelengths were selected for UV detection. Under the optimized extraction and chromatography conditions, ideal chromatograms were obtained which possessed not only abundant fingerprint information but also efficient separation characteristics including high signal responses, little interference and good peak shapes within 90 min (Fig. 3). Fig. 3 shows the typical chromatograms and ratio chromatograms of 27 batches of CBAT samples, as well as the chromatograms of the 5 marker standards.

### Chromatographic Fingerprint Analysis

#### Methodology Validation of Fingerprint Analysis

All the sample solutions as described in the section of ‘Sample and Standard Solution Pretreatment’ were used to perform the following experiments. The repeatability of the method was assessed by analyzing six independently prepared samples (S1) using the same experimental procedure. The precision of the instrument was determined by loading the same sample solution (S1) six times consecutively. The stability of the sample was examined by analyzing the same sample solution (S1) after 0, 3, 6, 12 and 24 hours.

The relative retention time (RRT) and relative peak area (RPA) of each co-possessing fingerprint peak were calculated to estimate the repeatability, precision and stability, and the results showed that, for repeatability, the relative standard deviations (RSD) of RRT and RPA were less than 0.5 and 4.8%, respectively; for precision, the obtained values did not exceed 0.3 and 2.7%, respectively; for stability, the obtained values were less than 0.3 and 2.6%, respectively. Thus, the validation results demonstrated that the method satisfied the fingerprint analysis criteria for CBAT samples.

#### Evaluation of Multi-wavelength Fingerprints

Reference fingerprint profiles (RFP) in both chromatograms and ratio chromatograms (Fig. 3) were generated from the mean of 27 sample chromatograms at corresponding wavelengths. The fingerprint and ratio fingerprint profiles of CBAT samples at 5 wavelengths (250, 276, 330, 360 and 375 nm) contained 39, 31, 15, 15 and 9 co-possessing peaks, respectively. In order to carry out HM quality control, the chromatograms with comprehensive chemical information characterization were required. However, the obtained fingerprint and ratio fingerprint profiles clearly showed that it was impossible to achieve this at a single analytical wavelength because of the complexity of the HM composition. Although the most abundant fingerprint information was acquired and the largest responses of saponins were displayed at 250 nm, weaker responses of the remaining constituents were also observed. Therefore, an appropriately integrated fingerprint assessment method capable of synthesizing both abundant fingerprint information and intense signal responses at 5 wavelengths was absolutely essential.

In this study, a novel integrated fingerprint assessment method based on information entropy was set up. Information entropy is a super-information characteristic digitized parameter of fingerprints, which reveals the total entropy value and absolute information amount of a fingerprint system [32]. Integrated assessment procedures were carried out as follows. Firstly, sample fingerprint signals at 5 wavelengths were imported into the TCM/HM fingerprint software so as to acquire five sets of evaluation parameters ($S_{\text{F}}^{\prime }$, *M*_F_ and *α* values, their RSD values were all below 3%, n = 3) of ratio fingerprints at 5 wavelengths (Table 2) and information entropy values (*S*_*j*_) of ratio fingerprints at the 5 wavelength. Secondly, three assessment parameters were combined according to Eq (9)–(11), where *e*_*j*_ represents the integrated weight at the *j*th wavelength and can be calculated based on Eq (8). $S_{\text{F}j}^{\prime }$, *M*_F*j*_ and *α*_*j*_, as well as $S_{\text{F}}^{\prime }$, $M_{\text{F}}^{\prime }$ and *α*′ (Table 2) represent three assessment parameters of the ratio fingerprints before and after integration, respectively. Finally, the integrated HM quality grades (Table 2) based on $S_{\text{F}}^{\prime }$, $M_{\text{F}}^{\prime }$ and *α*′ could be obtained according to TCM/HM quality criteria (Table 1) classified by SQRFM that could be used to comprehensively assess sample quality.

$$e_{j}=\frac{S_{j}}{\sum_{j=1}^{5}S_{j}}$$ (8)

$$S_{\text{F}}^{\prime }=\sum_{j=1}^{5}e_{j}{S^{\prime }}_{\text{F}j}$$ (9)

$$M_{\text{F}}^{\prime }=\sum_{j=1}^{5}e_{j}M_{\text{F}}{}_{j}$$ (10)

$$\alpha^{\prime }=\sum_{j=1}^{5}e_{j}\alpha_{j}$$ (11)

**Table 2:** The evaluation results of 27 CBAT samples by SQRFM.

**λ**	**Para.**	**S1**	**S2**	**S3**	**S4**	**S5**	**S6**	**S7**	**S8**	**S9**	**S10**	**S11**	**S12**	**S13**	**S14**
**250 nm**	$S_{\text{F}}^{\prime }$	0.95	0.98	0.94	0.96	0.99	0.97	0.98	0.98	0.98	0.98	0.97	0.96	0.98	0.98
	*M*_F_	84.0	96.8	101.2	92.1	93.8	101.3	95.2	95.8	104.1	97.2	99.3	100.6	97.6	94.7
	*α*	0.05	0.02	0.06	0.04	0.01	0.03	0.02	0.02	0.02	0.02	0.03	0.04	0.02	0.02
	Grade	3	1	2	2	2	1	1	1	1	1	1	1	1	2
**276 nm**	$S_{\text{F}}^{\prime }$	0.96	0.98	0.97	0.96	0.99	0.96	0.97	0.980	0.99	0.98	0.99	0.99	0.99	0.98
	*M*_F_	86.1	92.9	101.7	82.3	95.1	95.0	88.5	88.1	99.9	96.7	94.2	100.1	100.2	99.2
	*α*	0.05	0.02	0.03	0.04	0.01	0.04	0.03	0.02	0.01	0.02	0.01	0.02	0.02	0.02
	Grade	3	2	1	3	1	2	3	3	1	1	2	1	1	1
**330 nm**	$S_{\text{F}}^{\prime }$	0.97	0.99	0.97	0.98	0.99	0.99	0.99	1.	0.99	0.98	0.99	1.00	1.00	0.99
	*M*_F_	80.9	92.1	92.5	80.8	97.0	87.6	86.5	97.4	98.15	102.1	91.5	100.7	98.9	101.1
	*α*	0.03	0.01	0.03	0.02	0.00	0.00	0.01	0.00	0.005	0.02	0.01	0.00	0.00	0.01
	Grade	3	2	2	3	1	3	3	1	1	1	2	1	1	1
**360 nm**	$S_{\text{F}}^{\prime }$	0.97	0.97	0.85	0.98	0.98	0.98	0.99	0.98	0.97	0.98	0.97	0.98	0.99	0.99
	*M*_F_	81.2	86.9	90.0	91.3	96.8	90.3	85.2	90.2	85.6	96.6	82.0	90.1	99.2	98.0
	*α*	0.03	0.03	0.15	0.02	0.02	0.02	0.02	0.02	0.03	0.02	0.03	0.03	0.01	0.01
	Grade	3	3	4	2	1	2	3	2	3	1	3	2	1	1
**375 nm**	$S_{\text{F}}^{\prime }$	0.97	1.00	0.98	0.99	0.99	0.97	1.00	1.00	0.99	0.99	1.00	0.99	0.99	0.98
	*M*_F_	80.6	94.8	101.58	92.4	98.7	91.1	89.2	100.6	100.6	105.3	97.1	103.3	98.9	102.9
	*α*	0.03	0.00	0.02	0.01	0.01	0.03	0.00	0.00	0.01	0.01	0.00	0.01	0.01	0.02
	Grade	3	2	1	2	1	2	3	1	1	2	1	1	1	1
**Integrated**	$S_{\text{F}}^{\prime }$	0.96	0.98	0.94	0.97	0.99	0.98	0.98	0.99	0.98	0.98	0.98	0.98	0.99	0.99
	$M_{\text{F}}^{\prime }$	82.8	92.9	97.7	87.7	96.0	93.6	89.2	94.3	98.2	99.3	93.2	99.1	98.9	98.9
	*α*'	0.04	0.02	0.06	0.03	0.01	0.02	0.02	0.01	0.02	0.02	0.02	0.02	0.01	0.01
	Grade	3	2	2	3	1	2	3	2	1	1	2	1	1	1
***P***_**5C**_	–	0.86	0.99	0.93	0.97	1.02	0.96	0.97	1.02	1.02	1.01	1.02	1.06	0.98	0.96
**λ**	**Para.**	**S15**	**S16**	**S17**	**S18**	**S19**	**S20**	**S21**	**S22**	**S23**	**S24**	**S25**	**S26**	**S27**	**RFP**
**250 nm**	$S_{\text{F}}^{\prime }$	0.99	0.89	0.87	0.91	0.92	0.88	0.87	0.95	0.90	0.96	0.87	0.96	0.98	1
	*M*_F_	103.4	97.3	127.5	81.3	70.4	69.5	102.6	83.9	69.0	77.6	133.3	89.0	98.8	100
	*α*	0.01	0.11	0.14	0.09	0.08	0.12	0.13	0.05	0.10	0.04	0.14	0.04	0.02	0
	Grade	1	3	5	3	5	6	3	3	6	4	6	3	1	1
**276 nm**	$S_{\text{F}}^{\prime }$	0.98	0.90	0.86	0.91	0.87	0.91	0.89	0.96	0.91	0.96	0.85	0.97	0.98	1
	*M*_F_	107.2	103.9	116.4	82.0	71.1	89.2	109.9	85.9	72.3	79. 9	122.1	89.6	99.3	100
	*α*	0.02	0.10	0.14	0.09	0.13	0.09	0.11	0.04	0.09	0.04	0.15	0.03	0.03	0
	Grade	2	2	3	3	5	3	3	3	5	4	4	3	1	1
**330 nm**	$S_{\text{F}}^{\prime }$	0.99	0.98	0.97	0.89	0.99	0.88	0.96	0.99	0.96	0.99	0.96	0.99	0.98	1
	*M*_F_	95.2	93.9	185.1	81.0	79.1	82.4	148.1	78.5	72.9	81.1	190.3	88.5	95.9	100
	*α*	0.02	0.02	0.03	0.12	0.02	0.12	0.04	0.01	0.04	0.01	0.04	0.01	0.02	0
	Grade	1	2	8	3	4	3	7	4	5	3	8	3	1	1
**360 nm**	$S_{\text{F}}^{\prime }$	0.99	0.97	0.94	0.88	0.95	0.90	0.97	0.95	0.91	0.95	0.93	0.93	0.96	1
	*M*_F_	96.8	92.9	145.9	88.8	79.3	107.6	149.1	91.1	84.1	88.9	144.5	91.0	85.4	100
	*α*	0.01	0.03	0.06	0.12	0.05	0.10	0.03	0.05	0.09	0.05	0.07	0.07	0.04	0
	Grade	1	2	7	3	4	2	7	2	3	3	7	2	3	1
**375 nm**	$S_{\text{F}}^{\prime }$	1.00	1.00	0.95	0.84	0.97	0.85	0.96	1.00	0.96	0.98	0.95	0.99	0.99	1
	*M*_F_	97.8	97.2	168.8	83.1	73.1	84.4	134.0	76.7	72.1	82.0	172.8	81.1	97.6	100
	*α*	0.00	0.00	0.05	0.16	0.03	0.15	0.04	0.00	0.04	0.02	0.05	0.01	0.01	0
	Grade	1	1	8	4	5	4	6	4	5	3	8	3	1	1
**Integrated**	$S_{\text{F}}^{\prime }$	1.00	0.94	0.91	0.89	0.94	0.89	0.93	0.97	0.93	0.97	0.91	0.97	0.98	1
	$M_{\text{F}}^{\prime }$	100.5	97.3	147.0	83.1	74.3	85.8	126.7	83.4	73.8	81.6	151.2	88.1	95.8	100
	*α*'	0.01	0.06	0.09	0.11	0.07	0.11	0.08	0.03	0.07	0.03	0.09	0.03	0.02	0
	Grade	1	2	7	3	5	3	5	3	5	3	8	3	1	1
***P***_**5C**_	–	1.05	0.98	1.22	1.00	0.84	1.00	1.09	0.88	0.81	0.91	1.25	0.93	1.02	–

From Table 2, by observing the quality grades at 5 wavelengths and integrated wavelength, we found that: S1 and S13 had a constant quality grade at all wavelengths (grade 3 and 1, respectively); S17 and S25 had unqualified integrated grades (grade 7 and 8, respectively), while the remaining 25 samples had qualified integrated ones (grade 1–5); The integrated results exhibited some fluctuations and even greater differences compared with the quality grades at single wavelengths. For example, the integrated results of S3 and S22 were at medium level (grade 2 and 3, respectively) of grades (1, 2, 4 and 2, 3, 4, respectively) at single wavelengths. Interestingly, S17 and S25 exhibited unqualified integrated quality (grade 7 and 8, respectively) despite their having qualified grades (3, 5 and 4, respectively) at certain single wavelengths, conversely, S20 and S23 had qualified integrated quality (grade 3 and 5, respectively) in spite of their being unqualified (grade 6 and 6, respectively) at certain wavelengths. These grade changes might be attributed to differences in the fingerprint numbers and response strengths at different wavelengths, which illustrated that our evaluation strategy of combining multi-wavelength fingerprints was very comprehensive and essential to avoid any bias at a single wavelength.

In addition, the integrated $S_{\text{F}}^{\prime }$ and *α*′ values for 27 samples were all not below 0.89 and above 0.11, respectively, demonstrating that all samples had a similar chemical composition. Although they should be qualified and be in the range of grade 1–5 based on integrated $S_{\text{F}}^{\prime }$ and *α*′ from a qualitative perspective, in fact, S17 and S25 were judged as outliers (grade 7 and 8, respectively) in combination with integrated $M_{\text{F}}^{\prime }$ (147.0 and 151.2%, respectively) from a quantitative perspective, indicating that qualitative evaluation was first performed (an acceptable $S_{\text{F}}^{\prime }$ and *α*′ should not be below 0.7 and above 0.3, respectively) and then further quantitative assessment (an acceptable $M_{\text{F}}^{\prime }$ should be in the range of 70.0–130.0%) should not be ignored. Because the $M_{\text{F}}^{\prime }$ can reflect the overall ingredient content in a sample, it has the potential to be associated with medicinal efficacy in clinics. In our opinion, SQRFM which includes both qualitative and quantitative assessments should be a better method for TCM/HM quality evaluation, and it should provide a vital measurement strategy for ensuring TCM/HM safety and efficacy.

### Simultaneous Quantitative Analysis of the Five Marker Compounds

#### Methodology Validation of Quantitative Analysis

The linearity of the quantitative analysis method was assessed using a series of mixed standard solutions as described in the section of ‘Sample and Standard Solution Pretreatment’ under the optimized experimental conditions. The calibration curves were constructed by plotting the peak areas (*y*) vs. the injection masses (*x*, μg) of the 5 marker compounds. Their LOD and LOQ were determined by appropriately diluting the mixed standard solutions. The calibration curves, including the regression equations, correlation coefficients, linear ranges as well as LOD and LOQ, are displayed in Table 3. The correlation coefficients were all above 0.9996, indicating that the linear correlations of the 5 analytes were excellent between the peak area and injection mass over the tested ranges. According to the methodology validation experiments for fingerprint analysis, the peak areas of the 5 maker compounds were calculated for estimations of quantitative analysis repeatability, precision and stability, and the corresponding RSD values were found not to exceed 2.7, 1.2 and 1.6%, respectively. Recovery tests were performed using the standard addition method to evaluate the accuracy of the method and the mean recoveries of the 5 marker compounds were found to be between 96.0 and 104.0%. The above results demonstrated that the analysis method met quantitative requirements and was suitable for the simultaneous determination of ILQ, ILG, LQ, ILA and GLY in CBAT.

**Table 3:** Calibration plots, LOD and LOQ for the 5 compounds.

Compound	Calibration equation *y* = *kx* + *b* ^a^	*R*	**Linear range (μg)**	**LOD** ^**b**^ **(μg)**	**LOQ** ^**b**^ **(μg)**
**GLY/250 nm**	*y* = 666.52*x* + 35.826	0.9999	0.08–8	0.008	0.0272
**LQ/276 nm**	*y* = 1500.3*x* + 104.47	0.9997	0.055–5.5	0.0055	0.0187
**ILQ/360 nm**	*y* = 3604.1*x* + 91.002	0.9996	0.02–2	0.002	0.0068
**ILA /360 nm**	*y* = 2381.9*x* + 70.943	0.9998	0.025–2.5	0.0025	0.0085
**ILG/375 nm**	*y* = 7147.3*x* + 118.66	0.9998	0.02–2	0.002	0.0068

^a^ *y* and *x* were, respectively, the peak areas and masses (μg) of the analytes.

^b^ LOD was defined as the mass for which signal-to-noise ratio was 3 and the LOQ was defined as the mass for which the signal-to-noise ratio was 10.

#### Sample Analysis

The HM quality control in CBAT is carried out by determining the content of GLY in the Chinese Pharmacopoeia (2010). In fact, there are many other bioactive components besides GLY and, thus, the quantitative analysis of multiple components combined with chromatographic fingerprints was considered for quality control. The qualitative analysis of the 5 marker compounds in chromatograms were carried out by comparing the retention times and on-line UV spectra with those of marker standards to assign GLY, LQ, ILQ, ILG and ILA (Fig. 3). The developed HPLC method was applied to the simultaneous determination of the 5 marker compounds and the contents (mg/g) of the 5 marker compounds in 27 samples are summarized in S1 Table. From the obtained results, it was found that the contents of the 5 analytes varied markedly among the 27 samples, and this might be attributed to the raw herbs variability associated with a wide range of factors or variability in manufacturing processes.

**Fig 4:**
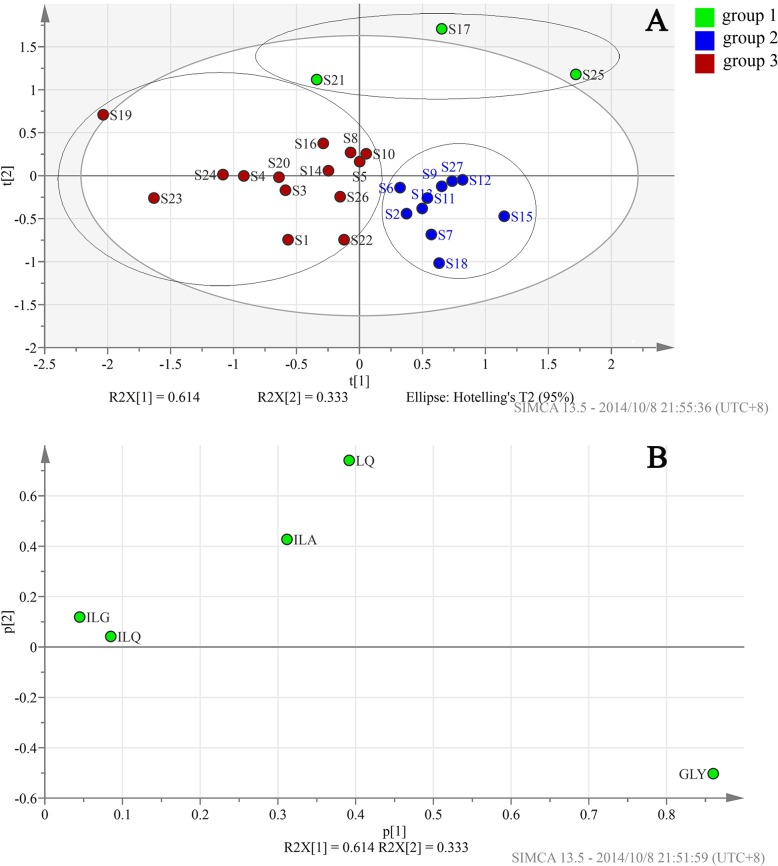
PCA score plot (A) and loading plot (B) of 27 batches of CBAT samples on the basis of the contents of the 5 marker compounds.

Principal component analysis (PCA) is an important chemometrics method [33] capable of reducing dimensions for high dimensional variables and, thus, it is helpful for simplifying and analyzing complicated problems. In order to investigate the differentiating ability of marker contents, PCA was conducted using SIMCA software (version 13.0) and the contents of the 5 marker compounds were used as input data to construct two-dimensional matrices (27 × 5), with 27 and 5 representing the sample number and marker type, respectively. A two-component PCA model was obtained which cumulatively accounted for 94.7% of the variation. The total variance explained for the first principal component was 61.4% and that for the second principal component was 33.3%, which was visible at the left bottom of the PCA score plot (Fig. 4A). In the loading plot (Fig. 4B), the coordinate position of each marker reflected marker weights in principal components and, so, the farther away from the coordinates-origin the marker position, the greater the correlation between the principal component and the marker. It was clear that GLY had the greatest correlativity with PC1, the same as LQ with PC2, and LQ and ILA had a greater correlativity with PC1, the same as GLY and ILA with PC2, while ILG and ILQ had less effect on PC1 and PC2. In the score plot, 27 batches of samples from the same manufacturer were distinctly divided into three clusters marked by group 1, 2 and 3, respectively. Three samples judged as unqualified (S17 and S25) and close to unqualified (S21) products by the integrated fingerprint assessment method fell into the same cluster (group 1), which was obviously different from the other two groups. In addition, the samples in group 2 had a positive correction with GLY, and the GLY contents (in the range of 19.992–23.387 mg/g) in group 2 were all higher than those (in the range of 10.377–19.65 mg/g) in group 3. Also, the samples in group 1 exhibited a positive correlation with LQ and ILA, hence the contents of LQ and ILA (in the ranges of 10.354–12.877 mg/g LQ and 3.669–4.839 mg/g ILA, respectively) in group 1 were all higher than those (in the ranges of 6.225–9.022 mg/g LQ and 3.639–1.599 mg/g ILA, respectively) in other two groups of samples. Consequently, it was found that the three products with the worst grades were clustered in the same group (group 1), mainly because of their high contents of LQ and ILA; the difference between group 2 and group 3 was mainly attributed to the contents of GLY. Therefore, we were able to conclude that the obtained two-component PCA model displayed better discriminating ability among the 27 samples from the same manufacturer.

#### Correlation between Corrected Quantified Ratio Similarity and Quantitative Analysis

In order to explore the relationship between the corrected quantified ratio similarity *M*_F_ values of ratio fingerprints and quantitative results of 5 marker compounds, data processing and analysis were performed as follows. Firstly, the *i*th marker compound contents (*z*_*i*_) in 27 samples were converted into percentage values (*P*_*i*_) of the marker contents out of its average content (${\bar{z}}_{i}$) of 27 samples as defined in Eq (12), and it was noteworthy that each sample weight should be corrected using a mass coefficient (*f*_*i*_) when calculating *P*_*i*_; Secondly, the mean of *P*_*i*_ values of the 5 marker compounds in each sample (i.e. *P*_5C_, Table 2) was calculated according to Eq (13), which was termed the logarithm-exponent mean.

$$P_{i}=\frac{z_{i}}{\bar{z}}f_{i}\times 100\%$$ (12)

$$P_{5\text{C}}=e^{\frac{1}{5}\sum_{i=1}^{5}\ln P_{i}}\times 100\%$$ (13)

**Fig 5:**
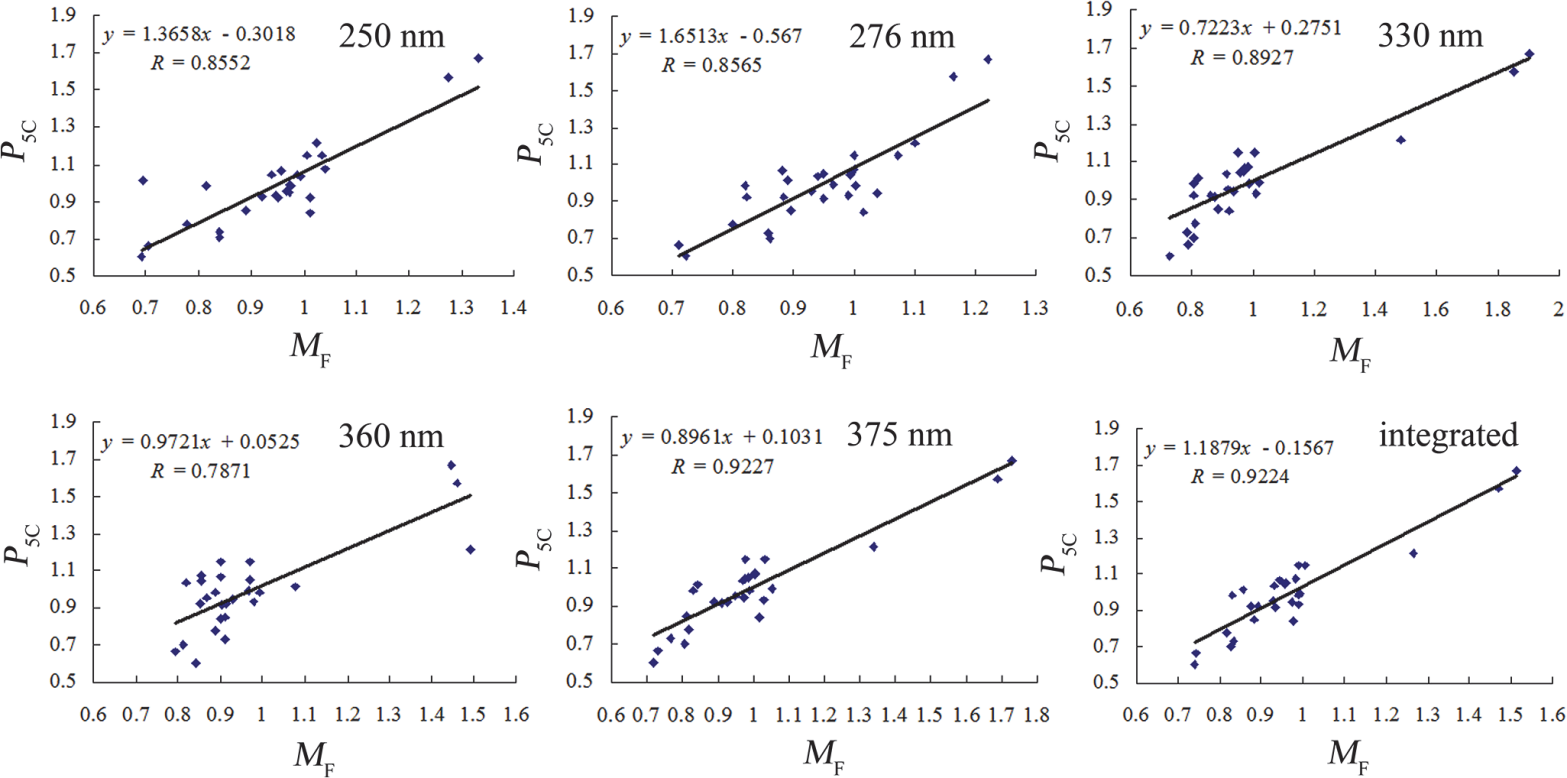
Correlation between *P*_5C_ and *M*_F_ for CBAT samples at 6 wavelengths. (A) 250 nm (B) 276 nm (C) 330 nm (D) 360 nm (E) 375 nm (F) integrated wavelength.

Finally, the *P*_5C_ values of 27 samples (*y*) were, respectively, plotted vs. the *M*_F_ values (*x*) at 6 wavelengths including integrated wavelength, 250, 276, 330, 360 and 375 nm (Fig. 5). From Fig. 5, it was apparent that there were better linear correlations between *P*_5C_ and *M*_F_ values at 6 wavelengths, because their correlation coefficients (*R*) were successively 0.9224, 0.8552, 0.8565, 0.8927, 0.7871 and 0.9227, which were all above 0.8500 except for 0.7871 at 360 nm and, especially at an integrated wavelength, reached a more satisfactory value of 0.9224. This indicated that the *M*_F_ values at the 6 wavelengths were basically consistent with the logarithm-exponent mean of the quantitative results for the 5 marker compounds. We concluded that multi-component quantification can be substituted by quantified fingerprint analysis for the purpose of TCM/HM quality control, which can be potentially applied to TCM/HM practical production due to its simplicity and economic advantage.

### Correlation Analysis between Quantified Fingerprints and Antioxidant Activities

CBAT contains many components with antioxidant activities, and the sample antioxidant capacity should take account of all the antioxidant components. In the present paper, the EC_50_ values of CBAT samples were used as the measurement parameter of antioxidant activity for 27 samples (Table 4). In order to explore the relationship between the quantified fingerprints and the antioxidant activities, attempts were made to correlate the EC_50_ to HPLC fingerprints at 250 nm and, as shown in S2 Fig., there were 28 out of 39 peaks in the fingerprints exhibiting negative correlation and 11 peaks had positive correlation with the EC_50_. PLSR, a well-known multivariate regression method [34,35], was also applied to explore the spectrum-effect relationship by taking into consideration the EC_50_ and the peak areas of 39 co-possessing peaks at 250 nm as two groups of variables. The regression characteristics were used to adjust a model to the measured data with the purpose of quantifying the relationship between two groups of variables. The appropriately adjusted model could be used to describe the relationship between the two variables and to predict new variables. The PLSR model was validated by means of full cross-validation for the purpose of rationality. From the relative errors (Table 4) of the predicted values from full cross-validation, two samples (S17 and S25) were identified as singular points, and thus they were eliminated when final mathematical model was constructed. Interestingly, two singular points, i.e. S17 and S25, were unqualified samples (grade 7 and 8, respectively) judged by the integrated fingerprint assessment method, indicating that the integrated quality grades on the basis of SQRFM were in agreement with the antioxidant activities of samples *in vitro*, while SQRFM as an TCM/HM fingerprint evaluation method and integrated multi-wavelength fingerprints as an assessment strategy were found to be both scientific and powerful. The obtained calibration model using the PLSR method is expressed by Eq (14), which shows that 22 out of 39 peaks in the fingerprints had greater correlation with EC_50_, and 14 peaks, including peak 1, 5, 7, 12, 15, 16, 18, 22, 23, 27, 28, 29, 30 and 35, were negatively correlated, while 8 peaks, including peak 4, 6, 17, 20, 21, 24, 34 and 38, were positively correlated with EC_50_.

**Table 4:** The measured and predicted EC50 values for 27 CBAT samples.

Sample	Measured EC_50_ (mg/ml, mean ± SD, n = 3)	RE of predicted EC_50_ from full cross-validation (%)	**Predicted EC**_**50**_ **(mg/ml)**	**RE** ^**a**^ **of predicted EC**_**50**_ **(%)**
**S1**	0.694±0.007	8.0	0.701	1.0
**S2**	0.641±0.006	0.1	0.639	–0.4
**S3**	0.585±0.005	8.5	0.580	–0.9
**S4**	0.661±0.008	16.1	0.673	1.8
**S5**	0.661±0.007	2.5	0.663	0.3
**S6**	0.771±0.009	–10.7	0.786	1.9
**S7**	0.679±0.008	5.7	0.690	1.6
**S8**	0.712±0.009	–6.2	0.702	–1.4
**S9**	0.684±0.006	–2.0	0.678	–0.8
**S10**	0.649±0.003	3.1	0.620	–4.5
**S11**	0.736±0.006	–7.4	0.710	–3.6
**S12**	0.721±0.007	–8.9	0.716	–0.8
**S13**	0.663±0.006	0.9	0.667	0.6
**S14**	0.663±0.008	5.1	0.674	1.6
**S15**	0.630±0.007	1.6	0.626	–0.6
**S16**	0.729±0.008	–8.0	0.736	1.0
**S17**	0.446±0.002	26.4	0.513	15.0
**S18**	0.632±0.006	–6.2	0.622	–1.6
**S19**	0.753±0.010	–6.7	0.768	2.0
**S20**	0.657±0.007	–5.2	0.676	2.9
**S21**	0.595±0.006	–1.5	0.603	1.3
**S22**	0.712±0.011	–12.4	0.680	–4.5
**S23**	0.826±0.012	–14.3	0.803	–2.8
**S24**	0.694±0.007	3.6	0.698	0.6
**S25**	0.522±0.005	–23.8	0.434	–16.9
**S26**	0.602±0.003	18.6	0.619	2.8
**S27**	0.585±0.005	11.6	0.595	1.7

^a^ RE was relative error.

$$\begin{array}{l} {\text{EC}}_{\text{5}0}=0.\text{9621}-0.000\text{5}x_{\text{1}}+0.00\text{112}x_{\text{4}}-0.00\text{115}x_{\text{5}}+0.000\text{54}x_{\text{6}}-0.00\text{199}x_{\text{7}}\\ -0.000\text{16}x_{\text{12}}-0.000\text{18}x_{\text{15}}-0.000\text{13}x_{\text{16}}+0.000\text{42}x_{\text{17}}-0.000\text{15}x_{\text{18}}+0.000\text{13}x_{\text{2}0}\\ +0.000\text{16}x_{\text{21}}-0.000\text{19}x_{\text{22}}-0.000\text{9}x_{\text{23}}+0.00\text{115}x_{\text{24}}-0.000\text{59}x_{\text{27}}-0.000\text{46}x_{\text{28}}\\ -0.000\text{21}x_{\text{29}}-0.00\text{188}x_{\text{3}0}+0.000\text{44}x_{\text{34}}-0.000\text{19}x_{\text{35}}+0.000\text{39}x_{\text{38}} \end{array}$$ (14)

**Fig 6:**
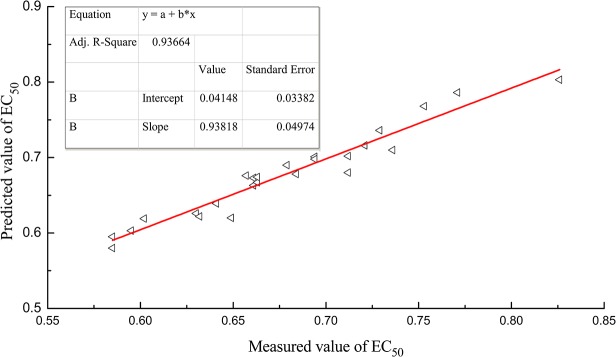
Correlation between the predicted and measured EC_50_ values for CBAT samples.

The above model was applied to predict the antioxidant activities of 27 batches of samples. The predicted EC_50_ values (Table 4) could be calculated using the areas of 22 peaks in the fingerprints correlated with EC_50_, and the absolute values of the relative errors of the predicted results (Table 4) were all less than 5% except for two singular samples (S17 and S25), indicating that the calibration model was robust. The calibration curve was also constructed by plotting the predicted EC_50_ (*y*) vs. the measured EC_50_ (*x*) of 25 batches of CBAT samples (except for S17 and S25), as shown in Fig. 6. We found that the calibration curve had a slope of 0.93818 (standard error, S = 0.04974) and an intercept of 0.04148 (S = 0.03382) at the left top of the plot, further indicating that the predicted EC_50_ values were coincident with the measured ones and the calibration model had an excellent predictive ability. Therefore, correlation analysis between the quantified fingerprints and the antioxidant activities can provide important medicinal efficacy information *in vitro* for HM quality control in CBAT.

## Conclusions

In the present study, a combination method of integrating multi-wavelength fingerprints and quantifying multiple bioactive components using HPLC-DAD was successfully developed and applied to 27 CBAT samples for the purpose of HM quality control. SRQFM for TCM/HM quality assessment was recommended, because it contains both qualitative and quantitative similarity evaluations and, thus, can overcome the deficiency of a single qualitative criterion and reflect the genuine quality of TCM/HM. The quality of 27 CBAT samples from the same manufacturer were well differentiated based on the integrated fingerprint assessment method of SQRFM, and two samples (S17 and S25) were judged as unqualified products (grade 7 and 8, respectively) and the remaining 25 samples were in the range grade 1–5. Moreover the correlation analysis between fingerprints and antioxidant activities was carried out using PLSR and the established model had excellent predictive ability, providing important information for CBAT quality control. This study offers a scientific, sensitive and comprehensive analytical strategy for the quality control of CBAT practical production.

## Supporting Information

S1 Fig The I values under different test conditions for CBAT samples. Extraction times: 20 min, 30 min and 40 min; Extraction solvents: methanol, ethanol and acetonitrile; analytical wavelengths: 250, 276, 330, 360 and 375 nm; column types: column 1, Century SIL C_18_ BDS (250 × 4.6 mm; 5.0 μm) and column 2, Agilent poroshell 20SB C_18_ (150 × 4.6 mm; 2.7 μm); mobile phases: mobile 1, water-glacial acetic acid (A; 100:0.2, v/v) and methanol-glacial acetic acid (B; 100:0.2, v/v), mobile 2, water-glacial acetic acid (A; 100:1, v/v) and acetonitrile-glacial acetic acid (B; 100:1, v/v), and mobile 3, water-phosphoric acid (A; 100:0.1, v/v) and methanol-phosphoric acid (B; 100:0.1, v/v).
(TIF)

S2 Fig The histogram of the Pearson correlation coefficients for the fingerprint peaks and the antioxidant activities. (TIF)

S1 Table The contents of 5 marker compounds for 27 CBAT samples. (DOC)
