# Supplementary figures and images for: Monitoring and Evaluating the Quality Consistency of Compound Bismuth Aluminate Tablets by a Simple Quantified Ratio Fingerprint Method Combined with Simultaneous Determination of Five Compounds and Correlated with Antioxidant Activities
Source: PLoS One. 2015 Mar 20;10(3):e0118223. doi: 10.1371/journal.pone.0118223 (PMC4368192; doi:10.1371/journal.pone.0118223)

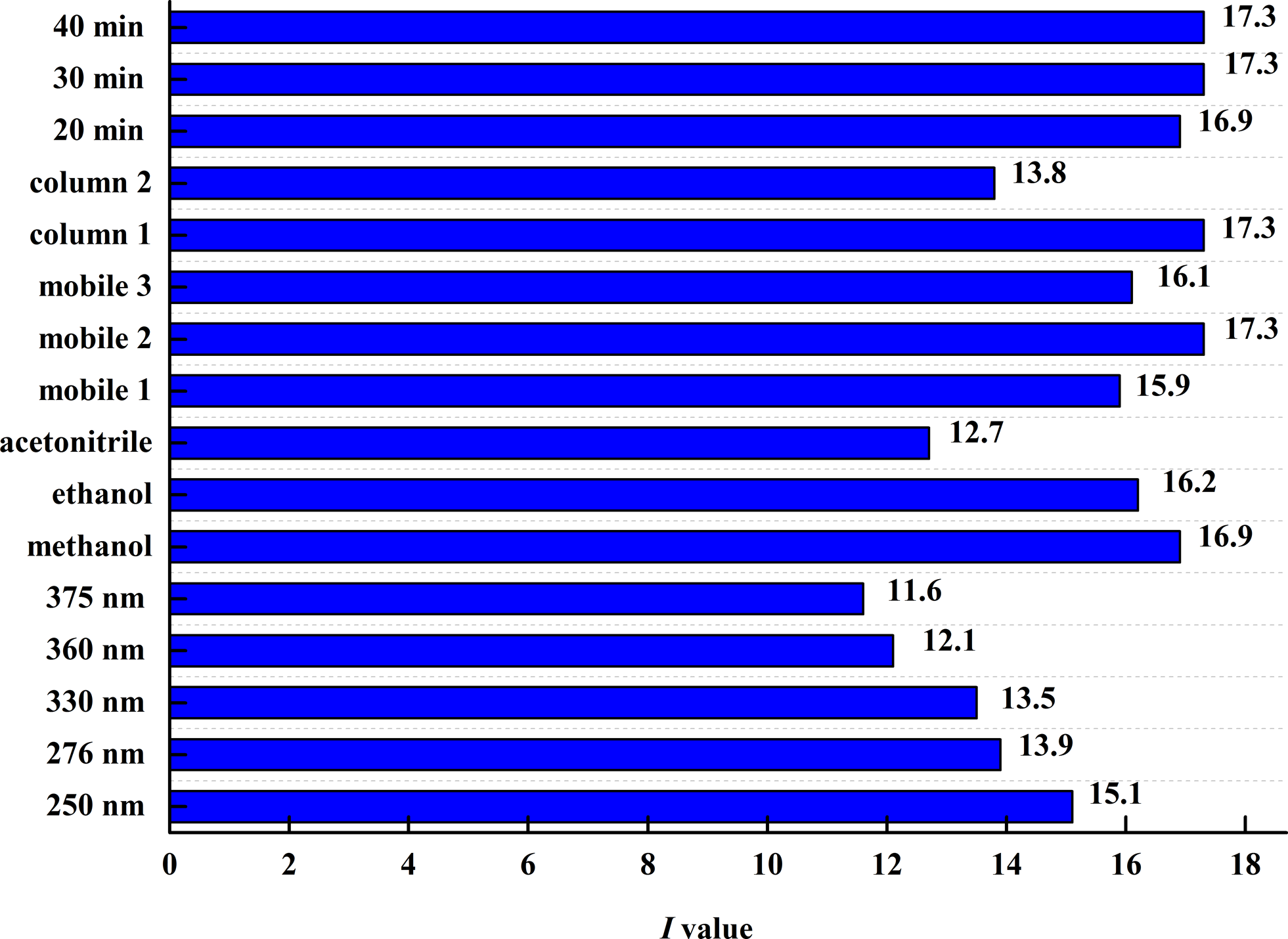

Supplement: S1 Fig — Extraction times: 20 min, 30 min and 40 min; Extraction solvents: methanol, ethanol and acetonitrile; analytical wavelengths: 250, 276, 330, 360 and 375 nm; column types: column 1, Century SIL C18 BDS (250 × 4.6 mm; 5.0 μm) and column 2, Agilent poroshell 20SB C18 (150 × 4.6 mm; 2.7 μm); mobile phases: mobile 1, water-glacial acetic acid (A; 100:0.2, v/v) and methanol-glacial acetic acid (B; 100:0.2, v/v), mobile 2, water-glacial acetic acid (A; 100:1, v/v) and acetonitrile-glacial acetic acid (B; 100:1, v/v), and mobile 3, water-phosphoric acid (A; 100:0.1, v/v) and methanol-phosphoric acid (B; 100:0.1, v/v). (TIF) [file pone.0118223.s001.tif]

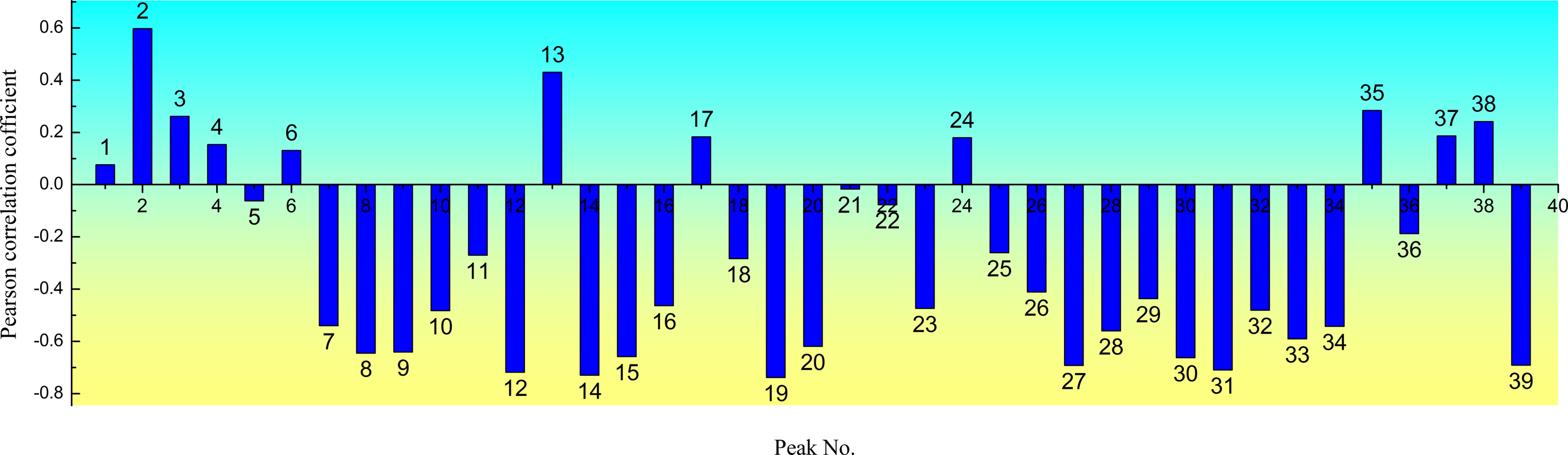

Supplement: S2 Fig — (TIF) [file pone.0118223.s002.tif]
