# Supplementary material for: Monitoring and Evaluating the Quality Consistency of Compound Bismuth Aluminate Tablets by a Simple Quantified Ratio Fingerprint Method Combined with Simultaneous Determination of Five Compounds and Correlated with Antioxidant Activities
Source: PLoS One. 2015 Mar 20;10(3):e0118223. doi: 10.1371/journal.pone.0118223 (PMC4368192; doi:10.1371/journal.pone.0118223)
Supplement: S1 Table — (DOC) [file pone.0118223.s003.doc]

| **Sample** | **Contents (mg/g, mean ± SD, n = 3)** | | | | |
| --- | --- | --- | --- | --- | --- |
|  | **GLY** | **LQ** | **ILA** | **ILQ** | **ILG** |
| **S1** | 18.229±0.045 | 6.348±0.044 | 1.902±0.030 | 0.159±0.003 | 0.078±0.001 |
| **S2** | 20.682±0.093 | 7.601±0.102 | 3.158±0.042 | 0.393±0.005 | 0.073±0.001 |
| **S3** | 16.926±0.073 | 7.143±0.080 | 2.758±0.049 | 0.330±0.006 | 0.062±0.000 |
| **S4** | 15.457±0.076 | 7.055±0.096 | 2.707±0.023 | 0.465±0.006 | 0.082±0.001 |
| **S5** | 18.222±0.088 | 8.458±0.070 | 3.417±0.049 | 0.432±0.007 | 0.093±0.001 |
| **S6** | 19.992±0.065 | 8.383±0.063 | 3.180±0.057 | 0.232±0.003 | 0.086±0.000 |
| **S7** | 21.876±0.110 | 7.492±0.124 | 2.858±0.046 | 0.287±0.003 | 0.085±0.001 |
| **S8** | 17.780±0.077 | 8.540±0.089 | 3.511±0.052 | 0.382±0.004 | 0.101±0.002 |
| **S9** | 21.032±0.064 | 8.652±0.108 | 3.534±0.035 | 0.311±0.002 | 0.107±0.002 |
| **S10** | 18.251±0.053 | 8.683±0.067 | 3.583±0.045 | 0.285±0.004 | 0.107±0.001 |
| **S11** | 20.923±0.076 | 8.200±0.083 | 3.348±0.027 | 0.353±0.005 | 0.104±0.002 |
| **S12** | 21.446±0.047 | 8.991±0.094 | 3.639±0.032 | 0.363±0.002 | 0.119±0.002 |
| **S13** | 21.076±0.086 | 8.257±0.133 | 2.868±0.043 | 0.314±0.004 | 0.082±0.001 |
| **S14** | 17.643±0.089 | 8.028±0.081 | 3.132±0.024 | 0.259±0.003 | 0.091±0.001 |
| **S15** | 23.387±0.067 | 8.654±0.071 | 3.313±0.051 | 0.405±0.007 | 0.105±0.001 |
| **S16** | 16.886±0.039 | 8.709±0.099 | 3.293±0.049 | 0.303±0.004 | 0.090±0.001 |
| **S17** | 17.399±0.108 | 12.544±0.105 | 4.839±0.019 | 0.706±0.009 | 0.206±0.003 |
| **S18** | 22.578±0.085 | 6.874±0.085 | 2.482±0.035 | 1.162±0.014 | 0.036±0.000 |
| **S19** | 10.377±0.096 | 7.554±0.044 | 2.444±0.037 | 0.128±0.001 | 0.083±0.001 |
| **S20** | 16.328±0.044 | 7.481±0.038 | 2.561±0.023 | 1.233±0.009 | 0.043±0.000 |
| **S21** | 15.270±0.088 | 10.354±0.124 | 3.669±0.064 | 0.492±0.003 | 0.148±0.001 |
| **S22** | 19.650±0.049 | 6.663±0.077 | 2.363±0.012 | 0.324±0.005 | 0.035±0.000 |
| **S23** | 13.700±0.134 | 6.225±0.056 | 1.599±0.023 | 0.112±0.001 | 0.088±0.001 |
| **S24** | 14.940±0.062 | 7.147±0.078 | 2.415±0.024 | 0.222±0.002 | 0.092±0.001 |
| **S25** | 22.082±0.019 | 12.877±0.116 | 4.740±0.037 | 0.729±0.005 | 0.210±0.003 |
| **S26** | 18.571±0.077 | 7.627±0.123 | 2.791±0.050 | 0.266±0.001 | 0.067±0.000 |
| **S27** | 21.221±0.033 | 9.022±0.101 | 3.485±0.020 | 0.325±0.004 | 0.091±0.001 |
